# Combination of ketamine and fentanyl (KetaFent) for safe insertion of ultrasound-guided central venous catheters in infants

**DOI:** 10.3389/fped.2023.1033793

**Published:** 2023-02-24

**Authors:** Vito D’Andrea, Giorgia Prontera, Giovanni Barone, Giovanni Vento

**Affiliations:** ^1^ Department of Woman and Child Health and Public Health, Fondazione Policlinico Universitario Agostino Gemelli IRCCS, Rome, Italy; ^2^ Neonatal Intensive Care Unit, Infermi Hospital, Rimini, Italy

**Keywords:** procedural pain management, pain measurement, analgesia, infant, ultrasound-guided central venous catheters

## Abstract

Centrally inserted central catheters (CICCs) are placed by ultrasound guided puncture of the internal jugular or brachio-cephalic vein. It is crucial to achieve a good level of sedation and analgesia in order to keep the babies still thus reducing the risk of procedural failure. The aim of this study is to evaluate the efficacy of the combination of ketamine and fentanyl during the CICC placement procedure. We retrospectively collected data from 72 infants who underwent CICC insertion after sedation with KetaFent protocol. The primary outcome was to assess the success of the procedure defined as CICC placement. Secondary outcome was intubation during the procedure in non-ventilated infants (noninvasive ventilation or spontaneous respiration), need for repeat doses of study medications to complete the procedure, time to complete the procedure, the level of analgesia assessed using vital parameters. The procedure was completed in 100% of cases. There were no cases of hypotension during and at the end of the procedure. No intubation was performed on non-ventilated infants. The combination of ketamine and fentanyl for sedation and analgesia in infants requiring insertion of a CICC is 100% successful. It is associated with a low risk of side effect like apnea and intubation. Insertion of a central venous catheter is a painful procedure for infants. Adequate sedation is mandatory to keep the baby still thus reducing the risk of procedural failure.

## Introduction

Power Injectable Central Venous Access Devices (CVADs) are the best venous access in critically ill infants. They allow high flow infusions (1 ml/s), easy administration of blood products, easy blood sampling and even haemodynamic monitoring. They can be placed even in preterm infants by ultrasound-guided puncture of the internal jugular vein or brachio-cephalic vein (centrally inserted central catheter or CICC) or femoral vein (femorally inserted central catheter or FICC) (1, 2); they are power- injectable 3–4Fr polyurethane catheters. Ultrasound-guided placement of CICC and FICC is feasible at any gestational age and birth weight (3–7), but is a painful procedure and it's mandatory to keep the babies still at the time of the venipunture in order to ensure the safety of the patients. Furthermore, pain has negative long-term neurodevelopmental consequences, such as cognitive impairment, learning disabilities, attention deficits, behavioral problems and motor abnormalities (8, 9). Currently, there is no international and national consensus on specific premedication before CVADs placement in neonates. An ideal drug for this context should have a rapid onset of action, subside quickly, provide good sedation and analgesia with the least possible adverse effects and, most importantly, have little effect on respiratory drive. In the NICU, infants are often given combinations of different analgesics and sedatives either simultaneously or sequentially to reduce potential side effectsThe aim of this study is to evaluate the efficacy of the combination of ketamine and fentanyl during the CICCs placement procedure.

## Material and methods

### Design and settings

We retrospectively collected data from 72 term and preterm infants who underwent CICC insertion after sedation with a combination of ketamine and fentanyl. This study was conducted in a level III NICU, at Policlinico Gemelli in Rome, Italy. Demographic and CVADs characteristics data were obtained from electronic medical records (Digistat®) and included gestational age at birth (GA), birth weight (BW), day of life and type of CVAD inserted, weight and mode of ventilation at the time of insertion, indication and site of insertion. We also recorded the duration of the procedure, body temperature before and after the procedure. Heart Rate (HR), FiO2, SatO2 and mean arterial blood pressure (MABP) were recorded 5 min before, during and 30 min after the end of the procedure. We obtained explicit informed consent from both parents for the purpose of this study.

### Outcomes

The primary outcome was to assess the success of the procedure defined as CICC insertion.

Secondary outcome was intubation during the procedure in non-ventilated infants (noninvasive ventilation or spontaneous respiration), need for repeat doses of study medications to complete the procedure, time to complete the procedure, level of analgesia using the vital parameters.

### Statistics methods

Continuous variables were expressed as mean ± Standard deviation (min and max) or as median (interquartile range) according to their distribution. Categorical variables were expressed as number and percentage. Student T test and Mann–Whitney U test were used to compare continuous variable, while Fisher's exact test was used to assess statistical significance in categorical variables. A *p* level < 0.05 was considered as statistically significant.

### CICC procedure insertion

All CICCs were inserted according to our insertion bundle for neonatal CVADs.

Preoperative ultrasound assessment of all central veins (“RaCeVA”: rapid central vein assessment) (10); maximal barrier protection measures; skin antisepsis with 2% chlorhexidine in 70% isopropyl alcohol; ultrasound-guided venipuncture of the brachio-cephalic vein (BCV) via the supraclavicular approach (visualisation in the long axis: in-plane puncture with linear “hockey stick” probe, 10–14 MHz); venous cannulation using the modified Seldinger technique and a micro-introducer kit; ultrasound assessment of the direction of the guidewire into the vasculature (ultrasound-based tip navigation) (11); tip location by intracavitary echocardiography (12); tunnelling of the catheter into the infraclavicular area; securing by the subcutaneously anchored sutureless devices (13); cyanoacrylate adhesive to close the puncture site and the exit site (14); covering the exit site with a semi-permeable transparent membrane. All procedures were performed at bedside, that is, in the incubator or in an open cot, according to the weight and postnatal age of the baby. Each patient was placed in a supine position, with a rolled sheet placed under the shoulders; the neck was in extension and the head turned slightly toward the opposite side of the chosen BCV. With this position, we were able to get a clear view of the BCV in all infants. During the procedure, the incubator was fully opened and the main operator was placed at the patient's head; a second operator was usually placed next to the main operator at the head of the baby managing the airways, especially if the baby was not intubated. A Neopuff Infant T-Piece Resuscitator was used if the baby became apneic during the deep sedation. An ultrasound screen was placed in front of the main operator at the opposite site of the incubator (15).

### Sedation protocol

The sedation protocol required the use of ketamine and fentanyl. Ketamine is often used for anaesthesia and analgesia in infants and children. It produces a dissociative state by blocking NMDA receptors, resulting in sedation, analgesia and amnesia, and has a short duration of action that supports haemodynamic and respiratory stability (16). It causes a slight increase in blood pressure and heart rate, a decrease in respiratory drive and mild bronchodilation (17) with minimal effects on cerebral blood flow (18). Despite these theoretical advantages, ketamine is a potent anaesthetic that has been little studied in neonates.

Fentanyl is a synthetic opioid commonly used in neonatal intensive care units. It is 50 to 100 times more potent than morphine (19). At IV analgesia occurs within 1 to 2 min, making it an ideal opiate for acute painful procedures. It does not cause as much haemodynamic instability as morphine because it does not cause histamine release (20). Therefore, it is better suited for neonates with hypovolaemia, haemodynamic instability, congenital heart disease or chronic lung disease. Fentanyl also reduces pulmonary vascular resistance and is useful in infants with persistent pulmonary hypertension (21). Chest wall rigidity is a known and serious adverse effect of bolus administration IV of fentanyl. However, this risk is mitigated by a slow IV infusion (over 5 min) and reversed by naloxone.

A bolus dose of 0.5 mg/kg Ketamine 10 min before the procedure was administered and Fentanyl in a slow intravenous bolus dose of 2 μg/kg over 5 min, 5 min before venipuncture. If the response was inadequate, further intermittent ketamine bolus doses of 0.5 mg/kg were administered every 10 min up to a maximum of 2 mg/kg (four boluses in total).

The nurse looking after the baby continuously monitored the infant during the procedure for adequacy of analgesia by assessing HR, SatO2, Fio2 and mean arterial blood pressure. This is part of our hospital policy for all invasive procedure. All date are recorded on papers and then copied into the electronic medical records (Digistat®).

During the procedure, the incubator was fully open and the main operator was in the right or left corner (depending on the venipuncture site). A second operator was usually near the main operator at the infant's head and took care of the airway, especially if the infant was not intubated. A Neopuff Infant T-Piece Resuscitator was used if the infant became apnoeic during deep sedation. If the infant developed haemodynamic instability, desaturations, respiratory arrest or bradycardia at any time, no further doses of ketamine were administered and supplemental oxygen was administered *via* a mask with a flow of 7–8 L/min and the need for supplemental oxygen was noted.

## Results

Between January 2020 and January 2022, we examined 72 infants. The mean gestational age was 32.1 ± 5 weeks. The mean weight at birth was 1762 ± 1000 g and the mean weight at insertion was *2526 ± 1000* g. The mean day of life at insertion was 52 (6–84). Of the infants included, 37 were mechanically ventilated, 6 received non-invasive ventilation and 29 breathed spontaneously. The indications for CICC placement are listed in Table 1. Regarding the site of placement 55 were placed in the right brachiocephalic vein and 17 in the left brachycephalic vein (23.6%). 93% were 3 Fr in size, 7% were 4 Fr in size.

**Table 1:** Baseline characteristics of enrolled infants (72 cases).

	72 cases
Gestational age at birth means ± SD (min-max)	32.1 ± 5 (24–41)
Birth weight means ± SD	1762 ± 1000
Weight at insertion means ± SD (min-max)	*2526.4 ± 1000* (630–5180)
Day of life at the insertion median (Q1–Q3)	52 (6–84)
Invasive ventilation *n* (%)	37 (51.4)
Non-invasive ventilation *n* (%)	6 (8.3)
Spontaneous respiration *n* (%)	29 (40.3)
**Indication *n* (%)**
Surgical disease	21 (29)
HIE	20 (27.8)
Prolonged parenteral nutrition	16 (22.2)
Infection	6 (8.3)
others	9 (12.5)
**CICC insertion site *n* (%)**
rBCV	55 (76.4)
lBCV	17 (23.6)
**CICC size *n* (%)**
3 Fr	67 (93)
4 Fr	5 (7)

Gestational age: weeks; Birth weight and weight at insertion: grams; n, numbers; HIE, hypoxia-ischemia encephalopathy; rBCV, right brachycephalic vein; lBCV, left brachycephalic vein.

We compared two populations of infants: 37 infants who received invasive mechanical ventilation and 35 infants who received non-invasive ventilation or spontaneous breathing. In both cases, the insertion of the CICC was successful, so the procedure was 100% successful in both populations. The mean time of the procedure was 23 min in infants with invasive mechanical ventilation and 25 min in the group of infants with spontaneous ventilation or non-invasive ventilation. No hemodynamic problems (hypotension or hypertension) were noted during the procedure or at the end of the procedure.

In the group of mechanically ventilated infants, an average of two doses of 0.5 mg/kg ketamine were administered; in the group of non-invasively ventilated or spontaneously breathing infants, an average of 3 doses of 0.5 mg/kg ketamine were administered.

Heart rate was recorded during the procedure. Analysis of heart rate change was evaluated during the most painful manoeuvres: venipuncture, tunneling, and insertion of the microintroducer. The average change was about 4 bpm in the intubated group and 5 in the non-intubated group. The temperature measured before the procedure and at the end of the procedure showed consistent values in both groups.

In the group of infants receiving invasive mechanical ventilation, 6 required supplemental oxygen; in the group of infants receiving spontaneous or non-invasive ventilation, 5 required supplemental oxygen. In the latter group, apnoea episodes were noted in only 2 infants and in no case was intubation required.

Two infants who received invasive mechanical ventilation and five infants who breathed spontaneously or received non-invasive ventilation received a maximum dose of ketamine. A median of 2 doses of ketamine were administered in the first group and 3 doses in the second group (Table 2). No significant differences were found in two groups.

**Table 2:** Study outcomes of enrolled infants.

	Invasive ventilation 37	Non-invasive ventilation Spontaneous respiration 35
**Primary outcome**
Success rate of CICC insertion	100%	100%
Duration of procedure minutes (min-max)	23 (17–32)	25 (20–41)
**Secondary outcome**
Dose of Ketamine median (Q1–Q3)	2 (1–2)	3 (1–3)
Infants achieving maximum dose of ketamine *n* (%)	2 (5.4)	5 (14.2)
*Δ* HR mean (min-max)	4 (2–5)	5 (2–10)
*Δ* BT mean (min-max)	0.6 (0–1.1)	0.7 (0–1.2)
Need for supplemental oxygen *n*	6	5
Apnea *n*	–	2
Need for intubation	–	–

n, number; HR, heart rate; BT, body temperature.

## Discussion

The results of this study suggest that the combination of ketamine and fentanyl is able to achieve a good level of sedation and analgesia necessary to keep the baby still for a safe insertion of the CICC.

CICC was successfully inserted in all patients. The insertion time was relatively short. In the non-intubated group, the insertion time was not statistically different from the intubated group, although the infants were not continuously sedated.

Temperature control was optimal during the procedure; this indicates both a fast procedure and optimal preparation before the procedure (thermal blanket and infant warmer).

Pain control in NICU is of paramount importance. In fact, several studies have shown that infants who have undergone multiple painful procedures have a worse neurological outcome (9). Pain in newborns occurs in the context of a developing brain with high plasticity that alters the normal development of the somatosensory system and subsequently the pain processing system that depends on sensory activity in early life (22). Both single and repeated painful procedures can have long-term consequences in term or premature infants (23, 24).

There are several pediatric pain scales that assess both behaviour, i.e., facial expression, crying, gross motor movements, and physiological indicators such as heart rate, blood pressure, etc (25–35). The most commonly used pain scales are Premature Infant Pain Profile-Revised (PIPP-R) (24, 26) and Neonatal Pain, Agitation, and Sedation Scale (N-PASS) (33). The first one evaluates the following parameters: heart rate, oxygen saturation, alertness, brow bulge, eye squeeze, nasolabial furrow; it is used for procedural and postoperative pain and can be used from week 26 of gestational age. N-PASS evaluates heart rate, respiratory rate, blood pressure, oxygen saturation, crying or irritability, behavior state, facial expression, extremities or tone; it is used for acute and prolonged pain and to assess sedation.

**Figure 1:**
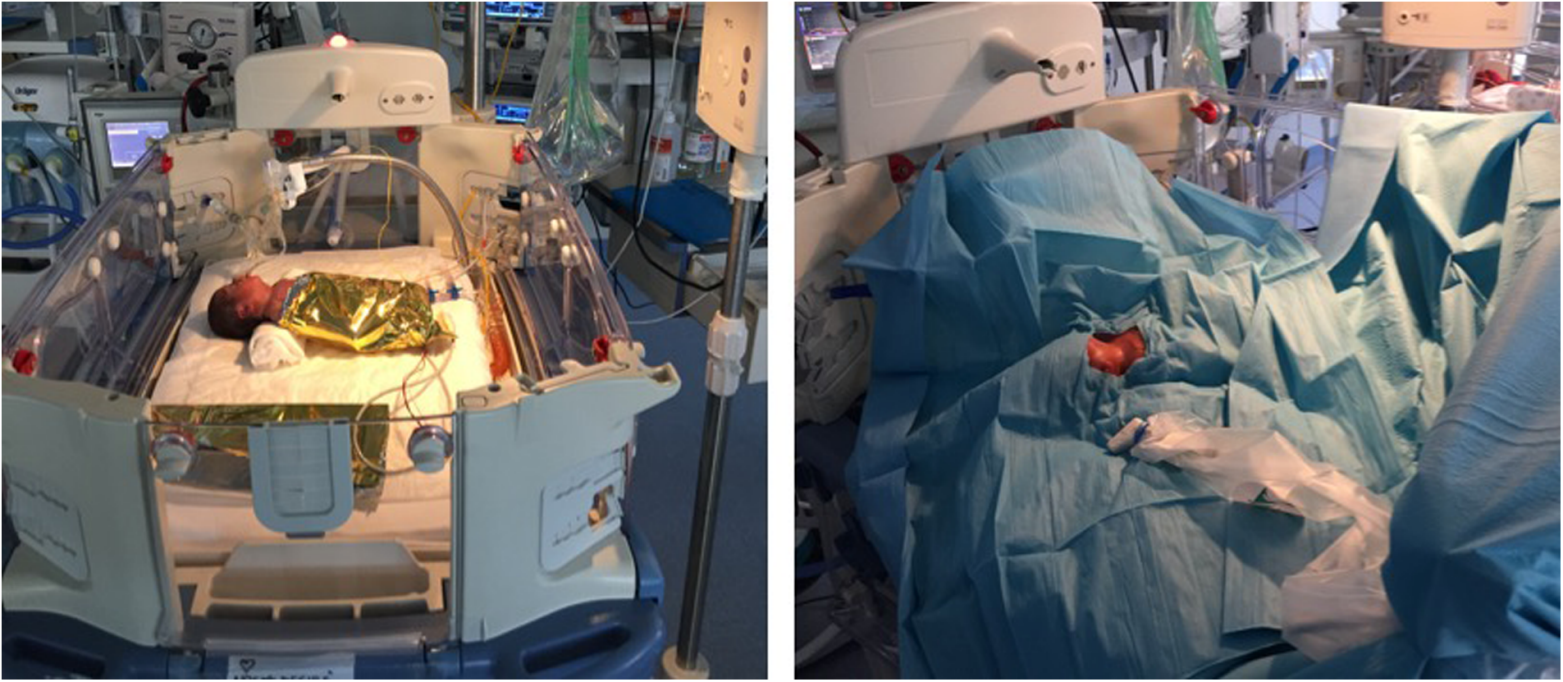
Pre-procedural setting: open incubator, the infant is wrapped in thermal blanket and then maximum barrier protection is used. The only visible area is the neck and chest area for the insertion procedure.

Unfortunately, in this study the infants were completely covered by sterile drapes and thermal blanket during the CICC insertion procedure (Figure 1). This was necessary to ensure maximal barrier precautions. Therefore, it was not possible to assess behavioural parameters such as eye sqeeze, nasolabial furrow or other facial expressions. Variations of vital parameters was then recorded and used as a proxy for pain control. Vitals recorded were within the normal range.

Fentanyl causes less histamine release than morphine and is therefore more suitable for neonates with hypovolemia, hemodynamic instability, congenital heart disease, or chronic lung disease. It offers equal level of analgesia. The most common side effects are chest wall rigidity, bradycardia and desaturation (35). Ketamine is widely used for anesthesia and analgesia in children but it is not well studied in neonatal population. It has a short duration of action, supporting hemodynamic and respiratory stability. Ketamine may also cause laryngospasm, respiratory depression, increased respiratory secretions and vomiting. 11 of 72 infants (15.2%) required supplemental oxygen and only 2 (2.7%) apnea episodes were recorded. In our population fentanyl and ketamine had a very good safety profile without any significant risk of apnea and desaturation.

The combination of fentanyl and ketamine was effective in our retrospective cohort to achieve a good level of sedation and analgesia for CICC insertion. This combination of drugs was associated with a low risk of apnea, hemodynamic instability and intubation. Unfortunately, pain cannot be fully assessed with our retrospective studies. Probably in the future tailored pain scale should be designed and adopted for pain assessment during CICC insertion. The findings of this study may be useful for research on this topic and should be interpreted cautiously pending results from future prospective trials.

## Data Availability

The original contributions presented in the study are included in the article/Supplementary Material, further inquiries can be directed to the corresponding author/s.
